# Functional Role of Intracellular Calcium Receptor Inositol 1,4,5-Trisphosphate Type 1 in Rat Hippocampus after Neonatal Anoxia

**DOI:** 10.1371/journal.pone.0169861

**Published:** 2017-01-10

**Authors:** Juliane Midori Ikebara, Silvia Honda Takada, Débora Sterzeck Cardoso, Natália Myuki Moralles Dias, Beatriz Crossiol Vicente de Campos, Talitha Amanda Sanches Bretherick, Guilherme Shigueto Vilar Higa, Mariana Sacrini Ayres Ferraz, Alexandre Hiroaki Kihara

**Affiliations:** 1 Laboratório de Neurogenética, Universidade Federal do ABC, São Bernardo do Campo, São Paulo, Brazil; 2 Departamento de Fisiologia e Biofísica, Instituto de Ciências Biomédicas, Universidade de São Paulo, São Paulo, São Paulo, Brazil; The University of Tokyo, JAPAN

## Abstract

Anoxia is one of the most prevalent causes of neonatal morbidity and mortality, especially in preterm neonates, constituting an important public health problem due to permanent neurological sequelae observed in patients. Oxygen deprivation triggers a series of simultaneous cascades, culminating in cell death mainly located in more vulnerable metabolic brain regions, such as the hippocampus. In the process of cell death by oxygen deprivation, cytosolic calcium plays crucial roles. Intracellular inositol 1,4,5-trisphosphate receptors (IP3Rs) are important regulators of cytosolic calcium levels, although the role of these receptors in neonatal anoxia is completely unknown. This study focused on the functional role of inositol 1,4,5-trisphosphate receptor type 1 (IP3R1) in rat hippocampus after neonatal anoxia. Quantitative real-time PCR revealed a decrease of IP3R1 gene expression 24 hours after neonatal anoxia. We detected that IP3R1 accumulates specially in CA1, and this spatial pattern did not change after neonatal anoxia. Interestingly, we observed that anoxia triggers translocation of IP3R1 to nucleus in hippocampal cells. We were able to observe that anoxia changes distribution of IP3R1 immunofluorescence signals, as revealed by cluster size analysis. We next examined the role of IP3R1 in the neuronal cell loss triggered by neonatal anoxia. Intrahippocampal injection of non-specific IP3R1 blocker 2-APB clearly reduced the number of Fluoro-Jade C and Tunel positive cells, revealing that activation of IP3R1 increases cell death after neonatal anoxia. Finally, we aimed to disclose mechanistics of IP3R1 in cell death. We were able to determine that blockade of IP3R1 did not reduced the distribution and pixel density of activated caspase 3-positive cells, indicating that the participation of IP3R1 in neuronal cell loss is not related to classical caspase-mediated apoptosis. In summary, this study may contribute to new perspectives in the investigation of neurodegenerative mechanisms triggered by oxygen deprivation.

## Introduction

The brain is an organ with a high energetic consumption using 20% of total body oxygen and 25% of glucose, and hence it is highly sensitive to oxygen reduction [1]. Oxygen deprivation leads to activation of several biochemical processes and may result in neuronal death through different biological processes, such as calcium influx, excitotoxicity, neuroinflammation and free radical production [2].

One of the brain regions more sensitive to oxygen deprivation is the hippocampus, a very well-studied structure related to spatial memory and learning [3]. Takada et al. demonstrated that neonatal anoxia induced alterations in rat hippocampal cells, such as different types of cell death, including apoptosis, necrosis, excitotoxicity, and maybe as a consequence, these rats present spatial memory deficits [4].

Previous studies have revealed the participation of intracellular calcium channels in neurodegeneration [5,6,7]. A study using NMDA-induced neuronal excitotoxicity demonstrated evidence that endoplasmic reticulum (ER)-Ca^2+^ release through ryanodine receptors (RyRs) and inositol 1,4,5-trisphosphate (IP3) receptors (IP3Rs) contributes to cell death. The inhibition of these receptors during the excitotoxicity insult suggests that calcium release by IP3R1 promotes mitochondrial dysfunction and ER-specific cell death pathway in neuronal excitotoxicity [6].

Calcium release from ER to cytosol follows non-linear dynamics according to the concentration of IP3Rs [8]. When a single IP3R is activated, the Ca^2+^ release is known as a ‘blip’ [9]. When IP3Rs are clustered, the receptors become more sensitive to both Ca^2+^ and IP3. This sensitivity is due to the increased probability of Ca^2+^ released from IP3R to bind to neighbour receptors, causing a cascade of opening of the receptors. Ca^2+^ released by the IP3R cluster is known as ‘puffs’ and several ‘puffs’ form waves [10,11]. The constant stimulation produces more regular spatiotemporal waves or oscillations [12,13], which present high potential to trigger and to synchronize cell activity [14]. Therefore, the distribution of IP3R along the organelles can influence Ca^2+^ release and, consequently, cell death induction.

To understand the participation of IP3R1 in neurodegeneration caused by anoxic insult, we investigated the distribution, subcellular localisation and functional role of IP3R1 in hippocampal cell death triggered by neonatal anoxia. This study is crucial and may provide valuable insights to develop therapeutic strategies to minimise the cognitive sequelae caused by oxygen deprivation.

## Materials and Methods

### Ethics statement

All animal studies were conducted in accordance with the recommendations in the Guide for the Care and Use of Laboratory Animals of the National Institutes of Health (NIH) and the Brazilian Scientific Society for Laboratory Animals. The protocol was approved by the Committee on the Ethics of Animal Experiments of the Universidade Federal do ABC (protocol number: 005/2014). All surgery was performed under anesthesia, and all efforts were made to minimize suffering.

### Animals

A total of 52 male Wistar rat pups, aged approximately 30 h and weighing 6–8 g, were used. The dams and the corresponding litters were housed in home cages at the vivarium of Universidade Federal do ABC, under 12:12 h light/dark cycle and at a constant temperature (23°C ± 1°C), with water and food provided *ad libitum*.

### Neonatal anoxia

Anoxia was induced in the 30-h-old rat pups as previously described by Takada et al. [15] with minor modifications. The animals were submitted to continuous flow (11.5 L/min) of 100% nitrogen (N_2_) for 25 min at 37°C in a non-hermetic chamber. Then, the pups were removed from the chamber, initialising the recovery process. The control group underwent the same procedure except for gas exchange, maintaining the airflow. After the exposure, the pups were returned to their dams, where they remained until the collection of material 24 h later.

### Real-time PCR

The hippocampi were dissected and immersed in 500 μL of TRIzol (Invitrogen, Carlsbad, CA, USA). The tissues were macerated and homogenised using a sonicator. Then, 100 μL of chloroform was added, and the resultant material was agitated by inversion and the sample was centrifuged for 15 min (10,000 rpm, 4°C). Next, 250 μL of isopropanol was added to the superficial translucent phase. The material was frozen at −20°C overnight for better aggregation of RNA. Then, the material was centrifuged for 10 min (10,000 rpm, 4°C), and the supernatant was discarded, preserving the precipitated RNA by addition of 1 mL of 75% ethanol and centrifugation for 5 min (7500 rpm, 4°C). The supernatant was discarded again, and the RNA sample was dried and then resuspended in 20 μL RNAse-free water. The RNA concentration was determined by spectrophotometry.

Real-time PCR (Corbett Robotics Inc., San Francisco, CA) was performed based on the SYBR Green system according to the specifications provided by the manufacturer. *IP3R1* gene expression was investigated using primer up 5′-CAGGCCGAGAGGAGGTGTGG-3′ and primer down 5′-GGGCAATCCCATGTCCGCGA-3′. *Cyclophilin A* gene expression was used as the internal control, using primer up 5′-GCGTTTTGGGTCCAGGAATGGC-3′ and primer down 5′-TTGCGAGCAGATGGGGTGGG-3′.

### IP3R1 and active caspase-3 immunofluorescence

For analysing IP3R1 immunofluorescence, the pups were perfused transcardially 24 h after the anoxic insult. The animals were deeply anesthetised using a mixture of ketamine and xylazine (75 and 10 mg/kg, respectively), and they were perfused with a pre-fixative solution consisting of 25 mM phosphate buffer (pH 7.0), 0.9% saline, 0.1% sodium nitrite and heparin (1 U/mL) at 4°C. Then, they were perfused with 1% PFA, consisting of 0.16 M phosphate buffer (pH 7.0), 1% paraformaldehyde and 0.2% picric acid at 4°C. Finally, they were perfused with phosphate buffer and 10% sucrose, and the brains were dissected and kept for 48 h in a cryoprotective solution of 30% sucrose at 4°C. After embedding in O.C.T. compound (Sakura Finetek, Torrance, CA, USA), the brains were cut at a thickness of 12 μm on the cryostat.

For analysing active caspase-3 immunofluorescence, the pups were perfused 24 h after intrahippocampal injection of 2-APB or vehicle. After deep anaesthesia and perfusion with intracardial 0.9% saline, a fixative solution composed of 4% paraformaldehyde was perfused. For both antibodies, the brain sections were dried at 37°C for 1 h. Antigen retrieval consisting of quenching by boiling 0.01 M citric acid solution (pH 6.0) for 7 min and cooling for 20 min was performed before analysing active caspase-3 immunofluorescence.

The slides were then washed in 0.1 M PB and placed in 0.5% blocking solution consisting of PBS + 0.3% Triton X-100 and normal donkey serum (NDS) for 40 min. After blocking, another washing was performed, and then the brain sections were incubated overnight with rabbit polyclonal antibody against amino acids 2732–2750 of rat IP3R1 (1:350, #2435031, AB5882, Millipore, USA)[16] or rabbit monoclonal antibody against residues subsequent to Ser29 of human Caspase-3 (1:250, #2365527, AB4-439, Millipore, USA)[17,18] in solutions containing 5% NDS and 0.5% Triton-X 100 in PBS 0.1M at room temperature. Then, the brain sections were washed and incubated with rabbit IgG tagged to Alexa 488 (1:500, A2120-6, Life Technologies, EUA), containing phosphate buffer + 0.3% Triton X-100 + DAPI, for 2 h at room temperature.

### Image acquisition and analysis

The brain sections were analysed under a fluorescence microscope (DM 5500, Leica Microsystems, Germany) coupled to a camera for image capture (DFC 365 FX, Leica Microsystems, Germany). Confocal images were captured in a 1024 × 1024 pixel format using a Zeiss LSM 780 confocal laser scanning inverted microscope (Carl Zeiss, Germany) at Centro de Facilidades de Apoio à Pesquisa, Universidade de São Paulo (CEFAP-USP). Image stacks comprised eight images captured with an LD Plan-Neofluar 40×/0.6 Korr M27 objective (Zeiss), applying a zoom factor of 1.5. Step intervals along the Z-axis ranged from 200 to 250 nm. Image processing and quantification of double-labelled structures were performed using the Zen 2011 software (Zeiss, version 11.00.190).

For the distribution analysis, three brain sections were selected with different levels of the hippocampus in each animal. Using the resources of the software Leica Application Suite Advanced Fluorescence (LAS AF, Leica Microsystems, Germany) for quantification, 18 standardised boxes (20 μm × 20 μm) were placed in the hippocampal sub-regions CA1, CA2-3 and the dentate gyrus, and the pixels relative to the positive labelling of IP3R1 were quantified. To evaluate the labelling co-localisation between the nucleus and IP3R1, three sections in five slides of each group were selected, using images under 40× objective magnification and through the Fiji software, an extension of ImageJ, the function Coloc 2 was used for the analysis of Manders’ overlap coefficient.

For cluster size analysis, images of coronal slices (n = 3) were transformed in binary matrices using the ImageJ software. We applied an intensity threshold determined for each image by comparing the binary matrix with the corresponding fluorescent image. After this primary treatment, the matrices were analysed using Matlab as follows: i) all clusters were identified and accounted for their size for each image and ii) plots from distribution probability densities were fitted into a log-normal function and mean cluster sizes were determined Eq (1).

The log-normal distribution is defined as

$$F\left(\text{ln}x,\mu ,\sigma \right)=\frac{1}{\sigma \sqrt{2\pi }}\text{exp}\left[-\frac{{\left(\text{ln}x-\mu \right)}^{2}}{2\sigma^{2}}\right],$$ (1)

where the parameters *μ* and *σ* are the mean and standard deviation of the associated normal distribution, respectively. The mean and variance of the log-normal distribution are defined as $e^{\mu +\sigma^{2}/2}$ and $\left(e^{\sigma^{2}}-1\right)e^{2\mu +\sigma^{2}}$, respectively.

### Bilateral hippocampal injection

Standard stereotaxic surgery technique was modified for neonates as previously described by Fitting et al. [19]. For this procedure, the pups were individually exposed to anoxia. After anoxia, the recovery process lasted 2 min, and the animals were cryogenically anesthetised. A low temperature was maintained during the surgery using cooled plates.

Rubber head bars were used to hold the skull in place when bilateral microinjections of vehicle (99.9% alcohol, n = 5) or 2-aminophosphate borate (2-APB, 20 μM concentration diluted in 99.9% alcohol, n = 5) were made directly into the hippocampus, more specifically in CA1/subiculum, using the following stereotaxic coordinates for injection: right hemisphere −0.3 mm anterior to the bregma, 0.7 mm medial to the bregma and −2.0 mm dorsal from the dura; left hemisphere −0.3 mm anterior to the bregma, −0.7 mm lateral to the bregma and −2.0 mm dorsal from the dura, using a 10-μL microsyringe (Hamilton Co., Nevada, USA). Then, 0.3 μL was injected on each hippocampus over 1 min after a 1-min resting period to allow the tissue to return to its original conformation and to prevent reflux. After the bilateral injections, the pups were kept in a warm condition under a heating mantle (35°C), until recovery of movements and reflexes, and were then returned to the dams.

2-APB is a blocker that has been introduced as an antagonist of IP3R [20]. Since then, several studies have used 2-APB to investigate the contribution of IP3Rs in cellular Ca^2+^ signal generation, and some studies revealed that 2-APB has different isoform selectivity, with remarkable effects on cells expressing types 1 and 3 of IP3R [21,22,23,24].

### Fluoro-Jade C assay (FJC)

After anaesthesia, the pups were transcardially perfused with 0.9% saline at 4°C, followed by 4% PFA in 0.1 M phosphate buffer (pH 7.4), at 4°C. The brains were dissected and kept overnight in 4% PFA solution and then changed to 30% sucrose solution for 48 h. After embedding in O.C.T. compound, the brains were cut at a thickness of 12 μm on the cryostat.

The FJC staining solution was prepared by adding 0.0005% of the stock solution to 99 mL of 0.1% acetic acid. The brain sections were washed in 0.1 M PBS and placed in 0.3 M Triton X-100 + DAPI solution for 2 h. After another wash, the brain sections were dried at 50°C for 1 h. Then, the sections were buffered in 99.9% alcohol and then in 70% alcohol and submitted to two washes by immersing in distilled water, each for 10 min. The sections were then placed in 0.06% potassium permanganate solution for 10 min and agitated gently. The slides were then rinsed three times for 10 min in distilled water, placed in FJC working solution (Millipore Corporate Headquarters, Billerica, MA, EUA) for 20 min and then washed three times in distilled water (each wash for 2 min) and dried in an oven at 50°C. After complete drying, they were immersed in xylene for at least 1 min and overlaid with coverslips using DPX.

### Terminal deoxynucleotidyl transferase (TdT)-mediated dUTP nick-end labelling (Tunel) assay

Histological analysis by Tunel technique is characterised by the incorporation of deoxyuridine trisphosphate fluorescein-12 (12-d-UTP) at the DNA 3′-OH ends, whose signal is amplified by the reaction involving the enzyme terminal deoxynucleotidyl transferase (rTdT), and the fragmented DNA marked by the 12-dUTP fluorescein becomes visible under the fluorescence microscope. We used the in situ cell death detection kit, TMR Red (Roche, USA), for coronal sections mounted on 12-μm-thick gelatinised slides (n = 5 per group). The slides were washed in 0.05 M PB and then incubated for 2 min in 1% sodium citrate solution in 0.05 M PB at 4°C. After additional washes in 0.1 M PB, 50 μL of Tunel reaction mixture was pipetted onto each slide. The slides were kept for 60 min at 37°C in the dark. After this procedure, they were washed again with 0.1 M PB, incubated in DAPI solution (1:65000) for 5 min, washed in 0.1 M PB, allowed to dry and then covered with glycerol. For IP3R1 and Tunel double labelling, we first conducted first immunofluorescence for IP3R1 followed by the Tunel assay.

### Statistical analysis

Two-way ANOVA followed by Tukey’s post hoc test was used for multiple comparisons of data with normal distribution to identify any significant differences between the treatment groups. For clusters analysis, the T-Student test was employed. All data were expressed as mean ± standard error of the mean (SEM).

## Results

### Neonatal anoxia decreases IP3R1 gene expression

**Fig 1:**
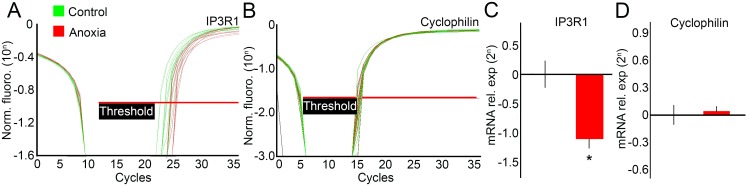
*IP3R1* gene expression in rat hippocampus 24 h after neonatal anoxia. We analysed *IP3R1* gene expression levels in the hippocampus of the control and anoxia groups using quantitative real-time PCR. Results were normalised by the mean of the control group and presented as fold-expression (2^*n*^). (A) Amplification plots from *IP3R1* gene expression in the control (green) and anoxia (red) groups. (B) Amplification plots from cyclophilin A gene expression in the control (green) and anoxia (red) groups, used as the internal control. (C) Quantification of gene expression of *IP3R1* (n = 8). A significant decrease in *IP3R1* gene expression (46%, *P < 0.001) was observed. (D) No differences in *cyclophilin A* gene expression could be detected between the control and anoxia groups.

Using specific primers designed for rat IP3R1, we observed that this gene is expressed in the hippocampus of neonates (control group, n = 8; anoxia group, n = 8). Notably, when compared with controls, the expression of IP3R1 in the hippocampus of neonates that suffered anoxia was statistically significantly decreased by 43% (2^-1.207 = 0.433 fold expression level, *P* < 0.001) (Fig 1).

### Neonatal anoxia does not change IP3R1 distribution in the hippocampus

**Fig 2:**
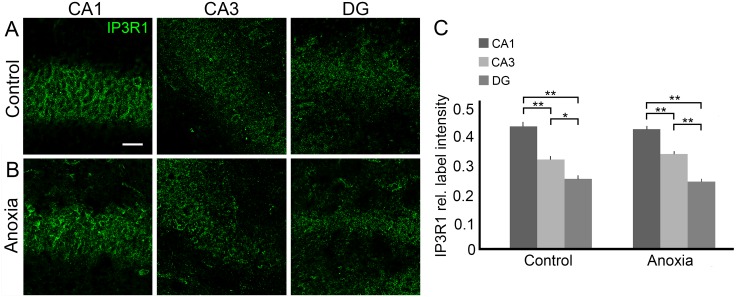
Distribution of IP3R1 immunolabelling in rat hippocampus 24 h after neonatal anoxia. (A) IP3R1 (green) distribution in CA1, CA3 and DG of control rat. (B) IP3R1 (green) distribution in CA1, CA3 and DG of anoxia rat. (C) Quantitative image analysis (n = 5) revealed that relative intensity is higher in CA1, followed by CA3 and DG, in both the control and anoxia groups. Scale bar: 25 μm. *P < 0.05; **P < 0.001.

Since we observed that anoxia decreased IP3R1 gene expression, we examined whether distribution of IP3R1 changes after this neurodegenerative insult. When we performed pixel density analysis of IP3R immunolabelling in the hippocampi of the control group (n = 5; CA1: 0.44% ± 0.03%; CA2-3: 0.32% ± 0.03%; GD: 0.24% ± 0.03%), we did not observe significant changes upon comparing these values with the group submitted to neonatal anoxia (n = 5; CA1: 0.43% ± 0.02%; CA2-3: 0.34% ± 0.02%; GD: 0.24% ± 0.03%). However, we were able to determine whether IP3R1 accumulated preferentially in CA1 followed by CA3 and DG in both the control and anoxia groups, and this result was important to determine the site of intrahippocampal injection (Fig 2).

### Neonatal anoxia triggers IP3R1 translocation and decreases mean cluster size

**Fig 3:**
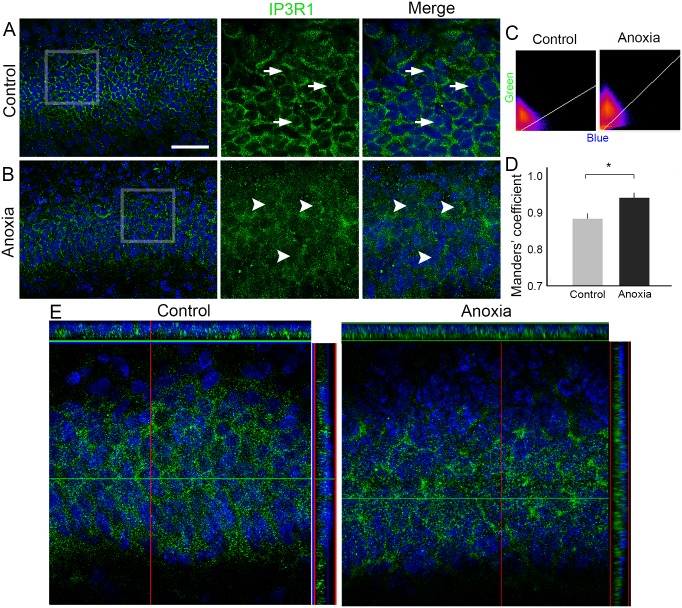
Subcellular analysis of IP3R1 distribution in rat hippocampus 24 h after neonatal anoxia. (A) Representative coronal sections of CA1 from control rats showing the localisation of IP3R1 labelling (green) and the nucleus labelled by DAPI (blue). Under high magnification of the selected area, it was possible to observe that IP3R1 labelling is typically located surrounding the cell nuclei in the control group (white arrows). (B) Representative coronal sections of CA1 subfield from rats submitted to anoxia showing the localisation of IP3R1 labelling (green) and the nucleus labelled by DAPI (blue). Under high magnification of the selected areas, it was possible to observe that IP3R1 labelling appears often to overlap the nucleus (white arrowheads). (C) Scattergrams represent the correlation between green and blue channels. (D) Manders’ coefficient analyses revealed that the overlap of green and blue channels is higher in the anoxia group than in the control group. (E) Confocal images of coronal sections with orthogonal axis confirming the differential distribution of IP3R1 labelling in the control (left) and anoxia (right) groups. Scale bar: 50 μm *P < 0.05.

Since we observed that neonatal anoxia does not change IP3R1 distribution in the hippocampal subfields, we performed a more detailed analysis considering the subcellular distribution of this protein. In the controls, IP3R1 labelling was observed as a punctate perinuclear pattern. On the other hand, in animals from the anoxia group, IP3R1 staining was observed as a diffuse, distributed punctate pattern, and labelling was often observed overlapping DAPI signals. Manders’ co-localisation analysis confirmed that compared to controls (n = 5; 0.876 ± 0.012), co-localisation of IP3R1 and DAPI labelling was significantly higher in the anoxia group (n = 5; 0.931 ± 0.010, *P* < 0.01) (Fig 3).

**Fig 4:**
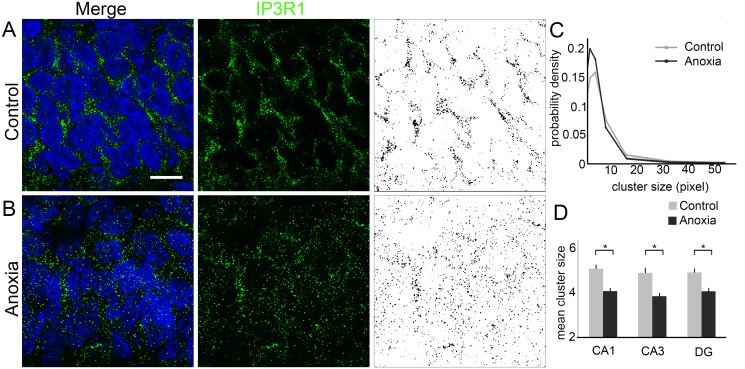
Cluster analysis of IP3R1 labelling in rat hippocampus 24 h after neonatal anoxia. (A) Representative high magnification image (100×) of CA1 from control rats showing the localisation of IP3R1 labelling (green) counterstained with 4′,6-diamidino-2-phenylindole (DAPI, blue). Binary image obtained from punctate labelling pattern of IP3R1 immunofluorescence. (B) Representative high magnification image (100×) of CA1 from rats submitted to anoxia showing the localisation of IP3R1 labelling (green) and the nucleus labelled with DAPI (blue). As obtained from controls, binary images were generated from punctate labelling pattern of IP3R1 immunofluorescence in the anoxia group. (C) Probability density function showing the cluster size in the control (grey) and anoxia (black) groups. (D) Graph representing the mean cluster size of the control (grey) and anoxia (black) groups in all the hippocampal subfields. Scale bar: 10 μm *P < 0.05.

Furthermore, we observed that anoxia triggered changes in the IP3R1-punctate labelling pattern. Based on the probability densities obtained from the images, we were able to fit a log-normal function to the data sets. Next, we obtained cluster mean sizes from the adjusted parameters. All the fits had high values of R^2^ parameter. When we compared the probability density of the cluster sizes, changes were detected after induction of anoxia. When compared with controls (n = 3), neonatal anoxia (n = 3) promoted significant decrease in the mean cluster size in all the hippocampal subfields. Mean values of the controls were higher than those of the anoxia group in CA1 (5.05 ± 0.77 vs. 4.06 ± 0.56, *P* < 0.05), CA3 (4.87 ± 0.97 vs. 3.83 ± 0.55, *P* < 0.05) and DG (4.89 ± 0.72 vs. 4.05 ± 0.62, *P* < 0.05) (Fig 4).

### Tunel-positive and Ip3R1 co-labelled cells did not alter after neonatal anoxia

**Fig 5:**
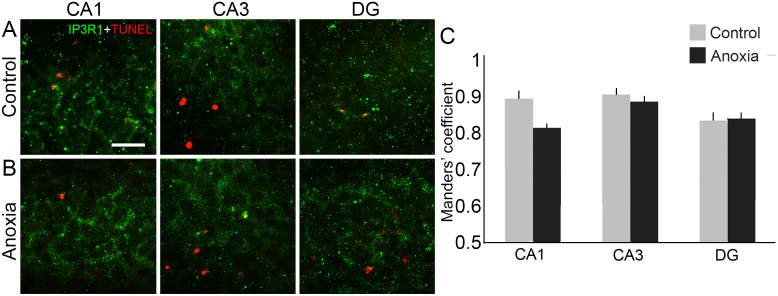
Double labelling of IP3R1 and Tunel staining after neonatal anoxia. (A) IP3R1 (green) and Tunel (red) double-labelling in CA1, CA3 and DG of control rat. (B) IP3R1 (green) and Tunel (red) double-labelling in CA1, CA3 and DG of anoxia rat. (C) Manders’ coefficient analyses did not show differences in the overlap of green and red channels in hippocampal subfields CA1 CA3 and DG when comparing control and anoxia groups. Scale bar: 25 μm.

The double labelling of IP3R1 and Tunel staining is not altered by neonatal anoxia, as shown by Manders’ co-localisation analysis (control, n = 2, CA1: 0.89 ± 0.02; CA3: 0.90 ± 0.02; DG: 0.83 ± 0.02; anoxia, n = 3, CA1: 0.81 ± 0.01; CA3: 0.88 ± 0.02; DG: 0.84 ± 0.01; Fig 5). This result suggests that apoptotic neurons maintain IP3R1 accumulation.

### IP3R1 blocker 2-APB decreases the number of FJC- and Tunel-positive cells in the hippocampus of animals submitted to anoxia

Since we were able to determine that our experimental model causes IP3R1 translocation to the nucleus and also decreases the mean cluster size, we aimed to study the role of IP3R1 in neurodegeneration triggered by anoxia. To this end, the study groups were divided into neonates submitted to anoxia and vehicle injection (VA, n = 5) and neonates submitted to anoxia and 2-APB injection (2-APB, n = 5).

**Fig 6:**
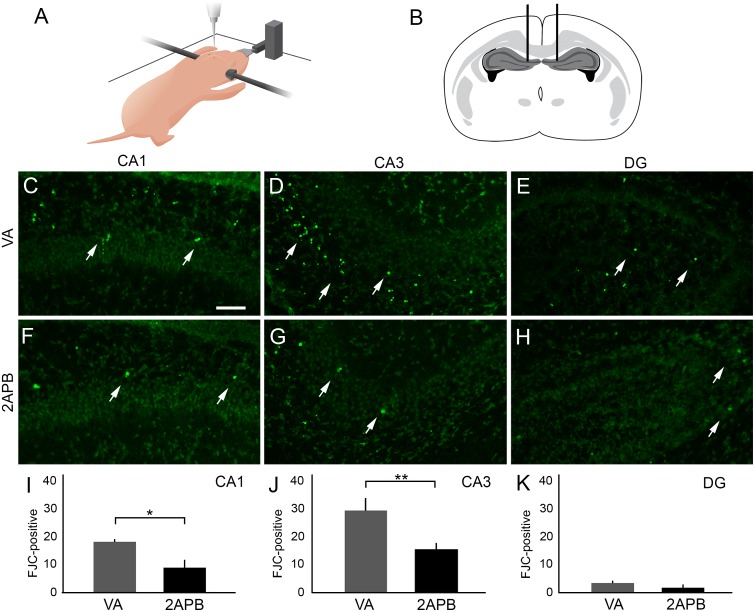
Distribution of Fluoro-Jade C (FJC)-positive cells in the hippocampus of rats submitted to anoxia and treated with 2-APB. (A) Schematic representation of a neonate rat positioned in a modified stereotaxic table. (B) Schematic representation of the bilateral intrahippocampal injection in the stereotaxic coordinates in the brain of a neonate rat. (C–E) Representative FJC staining (white arrows) in CA1, CA3 and DG coronal sections, respectively, from rat submitted to anoxia and injected with vehicle (vehicle anoxia, VA). (F–H) Representative FJC staining (white arrows) in CA1, CA3 and DG coronal sections from rat submitted to anoxia and injected with 2-APB (2-APB), respectively. (I) Graph showing the mean of FJC-positive cells located in CA1 from the VA and 2-APB groups. We observed decrease in FJC-positive cells in rats injected with 2-APB. (J) Graph showing the mean of FJC-positive cells located in CA3 from the VA and 2-APB groups. There was a decrease in FJC-positive cells in rats injected with 2-APB. (K) Graph showing the number of FJC-positive cells located in DG from the VA and 2-APB groups. Scale bar: 50 μm. *P < 0.05.

FJC staining in CA1 revealed significant decrease in the number of FJC-positive cells in the 2-APB group (8.83 ± 1.69) when compared with the VA group (*P* < 0.05). In CA3, when we compared the mean of FJC-positive cells in the VA and in 2-APB groups (29.4 ± 4.19 and 15.58 ± 1.76, respectively), we observed that 2-APB injection decreased the number of labelled cells after anoxia (*P* < 0.05). Interestingly, when we performed the same analysis on DG, we did not detect significant differences when we compared the number of FJC-positive cells in the VA and 2-APB groups (3.42 ± 0.75 and 1.75 ± 0.38, respectively) (Fig 6).

**Fig 7:**
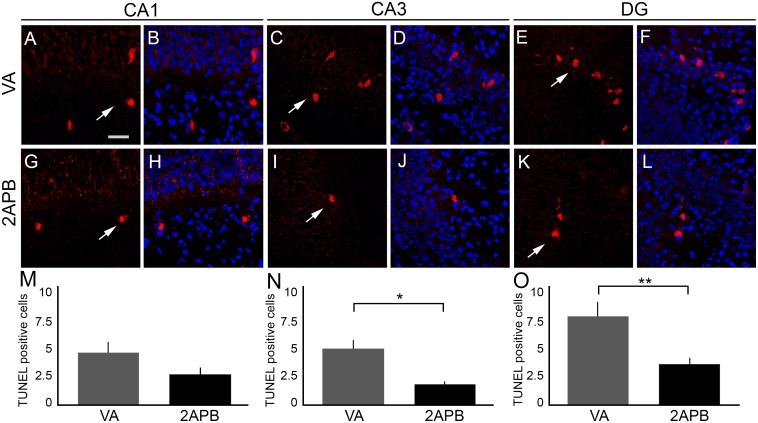
Distribution of Tunel-positive cells in the hippocampus of rats submitted to anoxia and treated with 2-APB. (A–B) Representative coronal sections of CA1 showing Tunel-positive cells (red, white arrow) in the hippocampus of rat submitted to anoxia and injected with vehicle (vehicle anoxia, VA) counterstained with DAPI (blue). (C–D) Representative coronal sections of CA3 showing Tunel-positive cells (red, white arrow) in the hippocampus of VA rat counterstained with DAPI (blue). (E–F) Representative coronal sections of DG showing Tunel-positive cells (red, white arrow) in the hippocampus of VA rat counterstained with DAPI (blue). (G–H) Representative coronal sections of CA1 showing Tunel-positive cells (red, white arrow) in the hippocampus of rat submitted to anoxia and injected with 2-APB (2-APB) counterstained with DAPI (blue). (I–J) Representative coronal sections of CA3 showing Tunel-positive cells (red, white arrow) in the hippocampus of 2-APB counterstained with DAPI (blue). (K–L) Representative coronal sections of DG showing Tunel-positive cells (red, white arrow) in the hippocampus of 2-APB counterstained with DAPI (blue). (M) Graph showing the number of Tunel-positive cells in CA1 of VA and 2-APB groups. We could not detect statistically significant differences between the groups. (N) Graph showing the number of Tunel-positive cells in CA3 of VA and 2-APB groups. There was a significative decrease in Tunel-positive cells in rats injected with 2-APB after neonatal anoxia. (O) Graph showing the number of Tunel-positive cells in DG of VA and 2-APB groups. We observed decrease in Tunel-positive cells in rats injected with 2-APB after neonatal anoxia. Scale bar: 25 μm. *P < 0.05. **P <0.01.

We next examined cell death by Tunel assay. We detected statistically significant differences when comparing the number of Tunel-positive cells from the VA and 2-APB groups in CA3 (5.00 ± 0.736 and 1.8 ± 0.255, respectively) and DG (7.875 ± 1.281 and 3.6 ± 0.557, respectively); but we did not observe differences in CA1 (4.625 ± 0.965 and 2.7 ± 0.604, respectively; Fig 7).

### 2-APB does not change the distribution of active caspase-3-positive cells in the hippocampus of animals submitted to neonatal anoxia

**Fig 8:**
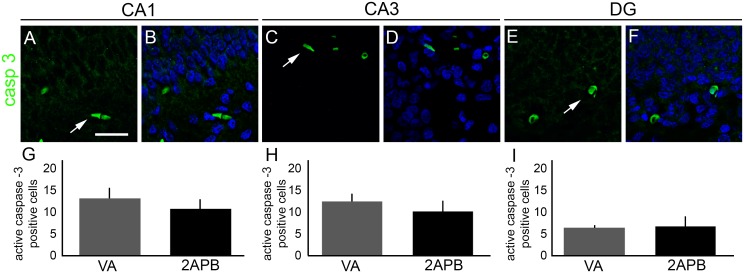
Distribution of active caspase-3-positive cells in the hippocampus of rats submitted to anoxia and treated with 2-APB. (A–B) Representative coronal sections of CA1 showing active caspase-3-positive cells (green, white arrows) of VA rats counterstained with 4′,6-diamidino-2-phenylindole (DAPI, blue). (C–D) Representative coronal sections of CA3 showing active caspase-3-positive cells (green, white arrows) in the hippocampus of VA rats counterstained with DAPI (blue). (E–F) Representative coronal sections of DG showing active caspase-3-positive cells (green, white arrows) in the hippocampus of VA rats counterstained with DAPI (blue). (G) Graph showing the pixel density of active caspase-3 immunofluorescence in CA1 subfield of VA and 2-APB rats. (H) Graph showing the pixel density of active caspase-3 immunofluorescence in CA3 subfield of VA and 2-APB rats. (I) Graph showing the pixel density of active caspase-3 immunofluorescence in GD subfield of VA and 2-APB rats. We could not detect statistically significant differences between the groups in all subfields. Scale bar: 50 μm.

After determining that 2-APB decreases the number of FJC and Tunel -positive cells after anoxia, we focused on the cellular pathway involved in this neuroprotection effect. For this purpose, the same groups used for FJC analysis were analysed for active caspase-3 immunofluorescence. Following vehicle or 2-APB intrahippocampal injections, we did not find statistically significant differences when comparing the number of positive cells for active caspase-3 from the VA and 2-APB groups in CA1 (10.60 ± 2.34 and 12.92 ± 1.88, respectively), CA3 (10.00 ± 1.67 and 12.25 ± 1.91, respectively) and DG (6.60 ± 0.48 and 6.33±0.87, respectively; Fig 8).

## Discussion

The hippocampus has been the subject of several types of research using different models of oxygen deprivation due to its susceptibility to insults [25]. Post-mortem analysis showed that the damage was almost entirely limited to the CA1 subfield of the hippocampus [26], whereas CA2 and CA3 subfields appear to be more resistant to ischaemic events [27,28]. In previous studies, our group showed that apoptosis occurs more in CA1 than in DG and CA2-3 subfields in an anoxia model as revealed by Tunel assay, although Fluoro-Jade B staining, a neurodegenerative marker [29], occurred preferentially in DG and CA3 [4]. Taken together, these results revealed that there are different types of cell death in the whole hippocampus after anoxia injury.

Neuronal loss caused by oxygen deprivation is conveyed by distinct biochemical processes, including excitotoxicity, neuroinflammation, free radical production and changes in calcium dynamics [2]. Indeed, cytosolic calcium appears to play essential roles in cell survival and physiology [30,31,32,33]. However, although intracellular receptors are key players in the dynamics of cytosolic calcium [34,35], there are very few studies related to deprivation of oxygen and intracellular calcium channels [36,37,38,39]. The present study has an unprecedented proposal, since there are no studies correlating the IP3R1 distribution and clusterization to the hippocampal cell death caused by neonatal anoxia in vivo model.

We observed that anoxia resulted in a decrease in IP3R1 gene expression after 24 h. In a study using non-differentiated PC12 cells, induction of apoptosis and increased expression of IP3R1 were observed after 3 h. However, 24 h after apoptosis induction, the authors observed a decrease of IP3R1 mRNA [40], corroborating our data. Another study showed that IP3R gene expression may vary according to the intensity and duration of oxygen deprivation stimulus and the age of the animal [41].

In addition to the decrease in gene expression, our mathematical analysis revealed decreased probability density of forming larger clusters after neonatal anoxia, which probably would interfere in the formation of long-lasting patterns as puffs and, consequently, in cell death induction. Taken together, the decrease in gene expression and the increase in the random distribution of IP3R1 might provide a cellular mechanism to prevent cell death caused by oxygen deprivation.

Besides we were not able to detect clear changes in IP3R1 distribution throughout the hippocampal regions after anoxia induction, immunostaining was more pronounced in CA1 than in CA3 and DG in both groups. The predominant IP3R1 accumulation in CA1 is consistent with previous studies performed in both young and adult mice [42,43,44].

On the other hand, accumulation of IP3R1 in the nucleus was increased after anoxia. These results are consistent with the study carried out *in vitro* with PC12 cells in which apoptosis triggered nuclear accumulation of IP3R1 [40]. These independent observations corroborate the reports about the presence of IP3Rs in the nuclear envelope and nucleoplasmic reticulum [45,46,47], suggesting that the nucleus also contains Ca^2+^ stores sensitive to IP3 [48,49]. Indeed, the consequences of Ca^2+^ signalling in the nucleus differ from those of cytosolic signalling, since nuclear signals may have effects on gene transcription [46]. It was postulated that during apoptosis, small nucleoplasm vesicles fuse to form large vesicles of the nucleoplasm and then more IP3Rs are translocated into the nucleus. In addition, during apoptosis, nuclear vesicles that store Ca^2+^ have an increased permeability, causing IP3R translocation to the nucleus, driving the transport of calcium through IP3R [40].

It has been shown that during apoptosis, Ca^2+^ released by ER-IP3R induced the liberation of cytochrome c by mitochondria, which translocates to the ER and binds to IP3R. This binding may result in a continuous open state of calcium channels and increased intracellular Ca^2+^ concentration in a sustained manner [50]. Based on these results, it is suggested that the increased sustained Ca^2+^ concentration constitutes one of the cell death signalling pathways, whereas Ca^2+^ oscillations convey physiological signals in the cell [50,51,52].

In the present study, to verify the role of IP3R1 in cell death caused by anoxia, 2-APB, a non-specific blocker of calcium release by IP3Rs, was injected and FJC technique was performed 24 h later to observe cell degeneration [53]. It was already reported that apoptosis induction increases the mRNA levels of Bax and caspase-3 [40]. However, when IP3R blocker was used in combination with apoptosis inducer, there was no increase in the levels of Bax and caspase-3, suggesting the involvement of IP3R in the caspase-mediated apoptosis. Indeed, it was proposed that IP3R1 in the cell nucleus is involved in the early process of caspase-mediated apoptosis and cluster formation in the nucleus [40].

Interestingly, in our study, 2-APB decreased the number of FJC- and Tunel -positive cells but did not alter the pixel density and distribution of active caspase-3. These results suggest that neuronal protection in the rat hippocampus after neonatal anoxia caused by the blockade of IP3R1 does not involve caspase-3 activity. In fact, stereological analysis of active caspase-3 using the same model did not show alterations [4].

The molecular mechanisms of IP3R1 involvement in cell death after neonatal anoxia remain to be elucidated. It seems that different types of cell death are present after this neonatal anoxia model and in some of them caspase-3 is not activated [4]. The caspase-independent cell death (CICD) occurs when a signal that normally induces apoptosis fails to activate caspases and usually requires upstream signalling pathways such as mitochondrial outer membrane permeabilization or activation of death receptor that can trigger a form of CICD termed necroptosis [54], a cell death type already described for neonatal hypoxia-ischemia model [55] and suggested to be present in this neonatal anoxia model [4].

Therefore, based on these results, it is suggested that IP3R1 participates in neuronal death processes in the rat hippocampus after neonatal oxygen deprivation, indicating the downregulation of gene expression 24 h after the stimulus, and although no difference was observed in the pixel density and distribution analysed by immunolabelling, we observed the translocation of IP3R1 to the nucleus. The presence of IP3Rs in the nucleus may influence various cellular functions, such as those of gene expression [46]. The present data, coupled with the finding regarding the difference in the probability of formation of different clusters sizes, suggest that neonatal anoxia promotes intense disruption in the subcellular distribution of IP3R1, which may have functional consequences.
